# An open-source, configurable machine learning pipeline for predicting blood culture outcomes from routine haematology parameters

**DOI:** 10.1099/acmi.0.001212.v3

**Published:** 2026-08-10

**Authors:** Benjamin R. McFadden, Timothy J. J. Inglis, Mark Reynolds

**Affiliations:** 1 University of Western Australia, School of Physics, Mathematics and Computing, Perth, 6009, Australia; 2 University of Western Australia, Discipline of Pathology and Laboratory Medicine, School of Medicine, Perth, 6009, Australia

**Keywords:** bacteraemia, blood cultures, machine learning, open-source software

## Abstract

**Background.** Bloodstream infections remain a major cause of morbidity and mortality worldwide, yet blood culture (BC) positivity rates are typically low, highlighting a need to optimize test use. Machine learning models trained on routinely available haematology parameters have shown promise for predicting BC outcomes. However, there is a lack of open-source software to implement methods described in the peer-reviewed literature.

**Results.** We present an open-source pipeline for training, evaluation, and reporting of binary classification models that predict BC outcomes from complete blood count (CBC), white blood cell differential (DIFF) and cell population data (CPD) generated by Sysmex XN-series haematology analysers. This pipeline implements four classifier types (LR: logistic regression, DT: decision tree, RF: random forest and XG: XGBoost), two default feature spaces (19-feature CBC/DIFF and 50-feature CBC/DIFF/CPD) and feature selection methods (Boruta all-relevant selection and recursive feature elimination), along with nested cross-validation (CV) to prevent data leakage during feature selection. Trained LR coefficients and DT rules were exported in portable formats suited to deployment in spreadsheet-based or laboratory information management system environments without requiring the Python programming language runtime. RF and XG models were also exported. The pipeline was fully configurable via a single JSON file and allowed adaptation to any binary classification problem without source code modification. Automated HTML reports with embedded area under the receiver operating characteristic curves and confusion matrices were generated for both training and inference runs.

**Conclusions.** This open-source repository addresses key limitations in existing BC outcome prediction workflows by providing a reproducible, transparent method and clinically deployable pipeline. Its configurable architecture, nested CV strategy, multiple feature selection methods and export of interpretable model artefacts make it suitable for both research and clinical decision support applications.

**Impact Statement**

This work provides an open-source, configurable machine learning pipeline that helps close the gap between methodological research and practical implementation for blood culture (BC) outcome prediction. By using routinely available haematology parameters, including complete blood count, white blood cell differential and Sysmex XN-series cell population data data, the pipeline supports the development of reproducible models that may assist diagnostic stewardship and more targeted use of BCs. Its inclusion of nested cross-validation and embedded feature selection reduces the risk of overly optimistic performance estimates, while automated reporting improves transparency and reproducibility. Importantly, the export of logistic regression coefficients and decision tree rules into portable formats makes the approach more accessible for deployment in spreadsheet-based, laboratory information systems and other low-infrastructure clinical environments. More broadly, its configurable design extends its value beyond BCs to other binary classification problems in infectious diseases and laboratory medicine.

## Data summary

 All the source code discussed in this paper is available at https://github.com/benjaminmcf/blood-culture-outcome-classification.

## Introduction

Bloodstream infections (BSIs) are a significant global burden on healthcare systems, contributing to prolonged hospital stays, excessive intravenous antimicrobial use and raised mortality [1, 2]. Blood culture (BC) is the standard of care for confirmation of BSI. However, BC is generally overused, with positive yields typically ranging from 5–15% [3, 4].

This low positivity rate results in unnecessary resource expenditure, delays in clinical decision-making while awaiting results and may contribute to unnecessary empirical antimicrobial exposure in some settings. Diagnostic stewardship, selecting the right test for the right patient at the right time, has therefore become an important strategy to optimize BC practice [5].

Machine learning (ML) approaches have emerged as a promising tool for diagnostic stewardship of BC, with examples in the literature leveraging the wealth of routinely generated data to predict BC outcomes before culture results are available [6–8]. However, their clinical, antimicrobial stewardship and antimicrobial resistance (AMR) impact depends on how predictions are implemented within local clinical and laboratory workflows [9]. One such example is the use of complete blood count (CBC) and white blood cell differential (DIFF) parameters, which are available within minutes to hours of blood collection. In addition, Sysmex XN-series haematology analysers report cell population data (CPD); fluorescence and scatter parameters that are used to characterize individual cell populations and report cellular abnormalities. These parameters provide a rich, multidimensional feature space for ML-based prediction.

In our previous work, we demonstrated the utility of an ML pipeline for BC outcome prediction using CBC/DIFF and CBC/DIFF/CPD feature spaces with retrospective data from Western Australia, achieving area under the receiver operating characteristic curve (area under the curve, AUC) scores of 0.75–0.76 on cross-validation (CV) and 0.76–0.82 on validation datasets [10].

Here, we present the software used in our previous work. The contribution of this work is not a new ML algorithm, but a reproducible, open-source, domain-specific implementation of established ML methods for BC outcome prediction. The novelty lies in packaging these methods into a configurable workflow designed for microbiology researchers, including leakage-aware evaluation, predefined haematology feature spaces, automated reporting and exportable artefacts that support deployment in low-infrastructure laboratory environments. The design choices presented in this paper are intended to reduce implementation burden, improve transparency and support reuse by microbiology and infection science researchers who may not otherwise have access to a complete end-to-end modelling workflow.

The Python analytical pipeline is open-source, implementing nested CV, feature selection, configurable target variables and feature spaces for generalizability, exporting of models, including logistic regression (LR) coefficients and decision tree (DT) rules in portable formats, and generating reports for both training and inference workflows.

## Theory and implementation

### Architecture overview

The software is available at https://github.com/benjaminmcf/blood-culture-outcome-classification. This software is implemented as an installable Python package with two command-line entry points*: bcoc-train* for model training and evaluation, and *bcoc-infer* for inference on new data. The codebase comprises four modules:

- *training.py*: Training entry point. Orchestrates configuration loading, data ingestion, nested CV, final model training, artefact exporting and report generation.
- *training_utils.py*: Core utilities. Contains configuration management, feature space definitions, model construction, feature selection [Boruta and recursive feature elimination (RFE)], nested CV, metric computation, plot generation and all input/output helpers.
- *inference.py*: Inference entry point. Discovers trained model artefacts via metadata JSON files, performs predictions using saved models or exported artefacts (LR coefficients, DT rules), computes performance metrics when ground truth is available, and generates an inference report.
- *reporting.py*: HTML report generation module for both training and inference workflows.

### Classification models

Four classifier types are implemented, encompassing both interpretable and ensemble methods:

- LR: A *LogisticRegression* classifier with *RobustScaler* preprocessing, wrapped in a scikit-learn *Pipeline* [11]. *RobustScaler* centres features by their median and scales by their interquartile range, which is more robust to outliers than standardization by mean and standard deviation. After training, the pipeline’s scaler parameters and classifier coefficients are algebraically collapsed into raw-space coefficients and an adjusted intercept. This produces a single set of coefficients that operate directly on unscaled feature values, enabling inference via a simple linear equation. This approach enables deployment using only arithmetic operations, making it suitable for spreadsheet-based or laboratory information system-integrated environments.
- DT: A *DecisionTreeClassifier* with balanced class weights and a maximum depth of 3. Decision rules are exported in both human-readable text format and structured JSON format, enabling deployment without a Python runtime.
- Random Forest (RF): A *RandomForestClassifier* ensemble of 100 DTs with balanced class weights, a maximum depth of 3 per tree, and out-of-bag scoring enabled [12].
- XGBoost (XG): An *XGBClassifier* with gradient-boosted trees [13]. The *scale_pos_weight* parameter is set to the class imbalance ratio (n_negative/n_positive) to handle the typically imbalanced BC dataset. One hundred estimators are used with a learning rate of 0.01 and a maximum tree depth of 3.

The default model parameters were selected to provide conservative, reproducible baseline models rather than optimized final models. For tree-based models, a maximum depth of 3 was chosen to limit model complexity, reduce overfitting risk, and improve the interpretability of exported decision rules. For RF and XG models, 100 estimators were used as a pragmatic default that provides ensemble stability while keeping training time and report generation manageable for users working on standard laboratory datasets. For XG, a learning rate of 0.01 was selected as a conservative shrinkage parameter so that individual trees contribute incrementally to the final model. These defaults are intended to support transparent baseline comparisons across feature spaces and feature selection methods. They should not be interpreted as universally optimal, and users may need to tune parameters using local validation data for specific datasets or deployment contexts.

### Feature spaces

The default configuration defines two feature spaces that reflect the parameters available on Sysmex XN-series analysers. CBC/DIFF (19 features) represents standard CBC with DIFF parameters, including absolute and percentage counts for white blood cells, red blood cells, haemoglobin, platelets, neutrophils, lymphocytes, monocytes, eosinophils, and basophils, as well as derived ratios, neutrophil-to-lymphocyte ratio (NLR), and monocyte-to-lymphocyte ratio (MLR). These features and their descriptions are provided in Table 1. CBC/DIFF/CPD (50 features) represents the CBC/DIFF feature set augmented with 18 additional CPD parameters (fluorescence, scatter, and position parameters for neutrophil, lymphocyte and monocyte populations) and 13 interpretive flags generated by Sysmex XN-series analysers (including flags for neutropoenia, neutrophilia, lymphopoenia, lymphocytosis, leukocytopoenia, leukocytosis, thrombocytopoenia and suspect flags for blasts, abnormal lymphocytes, left shift and atypical lymphocytes). These additional features and their descriptions are provided in Table 2.

**Table 1.** Complete blood count and differential features and their descriptions

Feature	Description
WBC (10^9^/L)	White blood cell count
RBC (10^12^/L)	Red blood cell count
HGB (g/L)	Haemoglobin
MCV (fL)	Mean corpuscular volume
MCHC (g/L)	Mean corpuscular haemoglobin
PLT (10^9^/L)	Platelet count
RDW-CV (%)	Red blood cell distribution width
NEUT# (10^9^/L)	Absolute neutrophil count
LYMPH# (10^9^/L)	Absolute lymphocyte count
MONO# (10^9^/L)	Absolute monocyte count
EO# (10^9^/L)	Absolute eosinophil count
BASO# (10^9^/L)	Absolute basophil count
NEUT% (%)	Neutrophil differential relative percentage
LYMPH% (%)	Lymphocyte differential relative percentage
MONO% (%)	Monocyte differential relative percentage
EO% (%)	Eosinophil differential relative percentage
BASO% (%)	Basophil differential relative percentage
NLR	Neutrophil to lymphocyte ratio
MLR	Monocyte to lymphocyte ratio

**Table 2.** Cell population data and interpretive flags along with their descriptions

Feature	Description
[NE-SSC(ch)]	Neutrophil complexity
[NE-SFL(ch)]	Neutrophil fluorescence intensity
[NE-FSC(ch)]	Neutrophil forward scatter
[LY-X(ch)]	Lymphocytes complexity
[LY-Y(ch)]	Lymphocytes fluorescence intensity
[LY-Z(ch)]	Lymphocytes size
[MO-X(ch)]	Monocytes complexity
[MO-Y(ch)]	Monocytes fluorescence intensity
[MO-Z(ch)]	Monocytes size
[NE-WX]	Width of dispersion of neutrophil complexity
[NE-WY]	Width of dispersion of neutrophils fluorescence
[NE-WZ]	Width of dispersion of neutrophils size
[LY-WX]	Width of dispersion of lymphocytes complexity
[LY-WY]	Width of dispersion of lymphocytes fluorescence
[LY-WZ]	Width of dispersion of lymphocytes size
[MO-WX]	Width of dispersion of monocytes complexity
[MO-WY]	Width of dispersion of monocytes fluorescence
[MO-WZ]	Width of dispersion of monocytes size
IP ABN(WBC)WBC Abn Scattergram	Abnormal clustering in the scattergram
IP ABN(WBC)Neutropenia	Presence of neutropoenia
IP ABN(WBC)Neutrophilia	Presence of neutrophilia
IP ABN(WBC)Lymphopenia	Presence of lymphopoenia
IP ABN(WBC)Lymphocytosis	Presence of lymphocytosis
IP ABN(WBC)Leukocytopenia	Presence of leukocytopoenia
IP ABN(WBC)Leukocytosis	Presence of leukocytosis
IP ABN(PLT)Thrombocytopenia	Presence of thrombocytopoenia
IP SUS(WBC)Blasts/Abn Lympho?	Suspected atypical lymphocytes or lymphoblasts
IP SUS(WBC)Blasts?	Suspected blasts
IP SUS(WBC)Abn Lympho?	Suspected abnormal lymphocytes
IP SUS(WBC)Left Shift?	Suspected presence of immature neutrophils
IP SUS(WBC)Atypical Lympho?	Suspected atypical lymphocytes

Both feature spaces and the target column are fully configurable via *config.json*, allowing the pipeline to be adapted to arbitrary binary classification problems without source code modification.

### Class imbalance handling

BC datasets are typically imbalanced, with negative cultures substantially outnumbering positive cultures. The pipeline, therefore, addresses the imbalance at two distinct stages: model training and prediction thresholding. During model training, balanced class weights are used for scikit-learn classifiers that support the *class_weight* parameter, so that errors in the minority class contribute more strongly to model fitting. For XG models, the analogous adjustment is implemented using *scale_pos_weight*, which is set to the ratio of negative to positive samples because this is the standard XG mechanism for weighting the positive class. These two approaches are, therefore, model-specific implementations of the same general principle.

In addition to training-time class weighting, the pipeline allows the probability threshold used to convert predicted probabilities into binary classifications to be configured at inference time. A threshold of 0.5 represents the conventional default for binary classification, whereas the default threshold of 0.3 favours sensitivity and negative predictive value, reflecting the clinical concern of avoiding missed positive BCs. This value of 0.3 was selected based on the results of our previous work [10]. However, this threshold should not be interpreted as universally optimal. In practice, the preferred threshold should be selected using local validation data and the intended clinical use case. We have also implemented the ability for users to select the classification threshold based on Youden’s index. Default behaviour of the software is to implement class weighting; however, this can be disabled so that only threshold tuning is implemented instead.

Because class imbalance can also affect the interpretation of predicted probabilities, model calibration may be required before clinical deployment, particularly if predicted probabilities are used directly for decision support rather than only for ranking or binary classification. Calibration methods, such as sigmoid or isotonic calibration are not applied by default in the current pipeline but represent an important area for future development and local validation.

### Feature selection

The pipeline implements two feature selection methods. Boruta all-relevant feature selection [14] is a wrapper around an RF model that iteratively compares real feature importance against shadow (randomly permuted) features. A feature is confirmed as relevant only if its importance consistently exceeds that of the best shadow feature across multiple iterations. The implementation uses a *RandomForestClassifier* base estimator with balanced class weights and a maximum depth of 3 [12]. The default Boruta parameters are used (maximum 100 iterations, significance level α=0.05). Features that are confirmed or tentatively accepted by Boruta are retained. RFE iteratively removes the least important features and rebuilds the model until a specified number of features remains. The implementation targets a compact subset of five features (or fewer if the original feature space is smaller). For pipeline-wrapped models (e.g. LR with *RobustScaler*), a *RandomForestClassifier* is used as the RFE base estimator since RFE requires direct access to feature importance or coefficient attributes. When feature selection is disabled (via *--fs none*), all features in the selected feature space are used, providing a baseline comparison. When *--fs all* is specified, the pipeline trains models with no selection, Boruta, and RFE, allowing direct comparison of all strategies. Because haematology parameters can be highly correlated, feature selection results should be interpreted cautiously. Correlated predictors may act as substitutes for one another, meaning that different CV folds may select different but clinically related features without necessarily indicating poor predictive performance. This is particularly relevant for feature-importance-based selection methods and for RFE, where the compact 5-feature subset may vary depending on the training partition. To make this visible to users, the pipeline records the features selected within each outer CV fold and summarizes feature-selection stability as the frequency with which each feature is selected across folds. These stability summaries are intended to support interpretation of the selected feature sets, but selected features should not be interpreted as stable biomarkers unless they are consistently selected and validated in independent datasets. To support interpretation of the final fitted models, the pipeline now includes post-training feature impact and correlation analyses. For supported models, Shapley additive explanations (SHAP) values are calculated for the final trained model to summarize the contribution of selected features to model predictions. These outputs are intended to help users identify which features have the greatest influence on predictions and whether their effects are consistent across samples. In addition, the pipeline generates a correlation analysis of the input feature space, allowing users to identify strongly correlated haematology parameters that may act as substitutes for one another during feature selection or model fitting. This is particularly relevant for CBC, DIFF and CPD variables, where biological and analytical relationships between parameters can result in substantial collinearity.

### Nested cross-validation

This pipeline implements nested CV to prevent optimistic bias from performing feature selection on the same data used for evaluation [15]. When feature selection methods, such as Boruta are applied to the full dataset before CV, selected features are influenced by the test fold data, leading to data leakage and inflated performance estimates. In the nested CV strategy employed by this pipeline, the outer loop performs stratified k-fold splitting. For each outer fold, the training partition (k-1 folds) is used for both feature selection and model training, while the held-out fold serves exclusively for evaluation. Feature selection is thus performed independently within each fold, ensuring that test-fold data never influence the feature selection process. The number of folds adapts dynamically to dataset size, targeting tenfold CV but reducing automatically when the minority class has fewer than ten samples per fold, as enforced by *StratifiedKFold* constraints. After nested CV provides unbiased performance estimates, a final model is trained on the entire training dataset. Feature selection is performed once on all samples (global selection), and the resulting model and feature list are saved as deployment artefacts. The nested CV metrics estimate the generalization performance of the overall procedure (feature selection and model training), not of this specific final model instance. The features selected within each outer fold are also retained, allowing the stability of selected features across CV folds to be summarized in the training report.

### Performance metrics

The pipeline computes a comprehensive set of binary classification metrics during both CV and inference: balanced accuracy, recall (sensitivity), specificity, precision (positive predictive value), AUC, Youden’s J statistic, F2 score (β=2, weighting recall over precision), positive likelihood ratio (LR+), and negative likelihood ratio (LR−).

### Artefact export and deployment

The pipeline is designed to bridge the gap between model development and clinical deployment. For each trained model, the following artefacts are produced:

- Model files (.*sav*): Serialized scikit-learn or XG model objects
- Feature lists (.*txt*): Selected features for each model configuration
- Model metadata (.*json*): Complete configuration, including model type, feature space, selection method, selected features and probability threshold
- LR coefficients (.*csv*): Raw-space coefficients enabling inference via arithmetic operations alone
- DT rules (.*txt, .json*): Human-readable and machine-parseable decision rules

The LR coefficients and DT rules are particularly significant for clinical deployment, as they can be integrated into existing laboratory workflows (e.g. Excel workbooks, laboratory information management systems, electronic decision support tools) without requiring any software installation.

### Validation of exported artefacts

The inference module includes a validation mode (*--validate*) that verifies exported artefact predictions match the original model object predictions, ensuring that the coefficient collapse and rule extraction processes introduce no numerical discrepancies.

### Automated reporting

The reporting module generates standalone HTML reports for both training and inference runs. Training reports include dataset statistics (total samples, class distribution), run configuration, a CV results table ranked by balanced accuracy, ROC curves per model (aggregated from nested CV out-of-fold predictions), and confusion matrices. Training reports also include a feature-selection stability summary, showing how frequently each feature was selected across the outer CV folds. This allows users to distinguish features that are selected consistently from those that may be selected only in particular training partitions. Where applicable, training reports also include feature impact and correlation outputs, including SHAP-based summaries of the final trained model and correlation heatmaps of the input feature space. These outputs are provided to assist interpretation of selected features and to highlight correlated predictors that may influence feature-selection stability. Inference reports are also produced, which include an input dataset summary, performance metrics tables, per-model prediction counts and positive rates, ROC curves and confusion matrices (when ground truth is available), and validation results when applicable.

### Configuration

All domain-specific parameters are externalized to a single *config.json* file. The *RANDOM_STATE* and *RANDOM_STATE_BORUTA* parameters are required and control reproducibility of model training and feature selection, respectively. The *TARGET_COLUMN* parameter (default: *isPOS*) specifies the binary outcome column, and *FEATURE_SPACES* (default: built-in *CBC_DIFF* and *CBC_DIFF_CPD* definitions) maps named feature sets to lists of column names. To adapt the pipeline to a different classification problem – for example, predicting urinary tract infection from urinalysis parameters – users need only modify *config.json*, without editing the rest of the source code.

### Software dependencies

The software requires Python≥3.11 and depends on the following packages: scikit-learn (≥1.70.2) [11], XG (≥3.00.5) [13], Boruta (≥0.40.3) [14], pandas (≥2.30.3) [16], NumPy (≥2.30.4) [17], matplotlib (≥3.100.7) and imbalanced-learn (≥0.140.0). The project is distributed as a standard Python package with a *pyproject.toml* build configuration.

### Prevalence sensitivity analysis

Because BC positivity varies across settings, the pipeline supports the evaluation of model behaviour across assumed positive class prevalences from 5–15%. Using the nested CV out-of-fold predictions, the pipeline recalculates prevalence-dependent metrics under each assumed prevalence while preserving the observed sensitivity and specificity at each threshold. This analysis is performed for the default threshold and the Youden-selected threshold. Metrics reported include positive predictive value, negative predictive value, F1 score, balanced accuracy, false-positive rate, and false-negative rate. Results are presented as line plots and accompanying tables to show how clinical utility varies across plausible deployment prevalence levels. This analysis is not intended to replace external validation in populations with different positivity rates, but provides a transparent estimate of how prevalence-dependent metrics would be expected to vary across clinically plausible base rates.

### Pipeline utilities

A cleanup script (*clean.py*) removes all pipeline output directories, enabling a complete end-to-end test cycle. Additionally, there is an example Jupyter notebook that demonstrates how to load exported LR coefficients and DT rules, compute predictions and validate them against the original model objects.

## Discussion

### Avoiding data leakage in feature selection

The pipeline implements nested CV. In some cases, feature selection may be performed on the entire training set before stratified tenfold CV, meaning that selected features were influenced by samples that later appear in test folds. This is a well-documented source of optimistic bias in ML evaluations [15, 18]. By moving feature selection inside the CV loop, this pipeline produces unbiased estimates of generalization performance that more accurately reflect the expected performance on truly unseen data.

### Clinical deployment

A key design goal is to enable deployment of trained models in clinical environments that may not support Python-based inference. The algebraic collapse of scaled LR pipelines into raw-space coefficients produces a single set of weights and an intercept that can be applied using basic arithmetic; a capability that is directly applicable to spreadsheet-based workflows common in clinical laboratories. Similarly, the export of DT rules in both human-readable text and structured JSON formats allows decision logic to be implemented in any programming language or rule engine. The *--validate* flag provides confidence that these exported artefacts produce identical predictions to the original model objects. The RF and XG models are still exported and could be deployed given the appropriate infrastructure.

### Feature selection strategies

The inclusion of both Boruta and RFE provides complementary feature selection strategies. Boruta identifies all features that carry information relevant to the outcome, tending to retain larger feature sets. RFE, by contrast, selects a compact subset (default: five features), which may be more practical for clinical deployment where a small number of predictors is preferred. Running both methods (via *--fs all*) allows researchers to compare the trade-off between model complexity and predictive performance. The addition of SHAP and correlation analyses provides users with further context for interpreting selected features. SHAP summaries can indicate which features contribute most strongly to the final model’s predictions, while correlation heatmaps help identify groups of related haematology variables that may be selected interchangeably. However, these outputs should be interpreted as model explanation tools rather than evidence of causal relationships or definitive biomarker importance. In particular, when predictors are highly correlated, feature attribution may be distributed across related variables or may vary depending on the selected model and training data. Therefore, SHAP and correlation outputs should be used alongside feature-selection stability, clinical plausibility and external validation.

### Configurability and reuse

Another design decision of this pipeline is to avoid tightly coupling it to the BC outcome classification problem by using hardcoded feature names and target variables. This pipeline externalizes all domain-specific parameters to the *config.json* file. This design decision enables researchers working on related binary classification problems in infectious diseases, such as predicting urinary tract infections from urinalysis parameters, AMR from laboratory data or sepsis from physiological measurements, to reuse the pipeline’s architecture, nested CV strategy and reporting capabilities without modifying source code.

### Automated reporting

The generation of standalone HTML reports for both training and inference runs improves reproducibility and facilitates communication of results. Training reports provide a complete summary of the CV experiment, including embedded ROC curves and confusion matrices generated from aggregated out-of-fold predictions. Inference reports summarize model performance on test data and document the distribution of predictions, enabling assessment of model behaviour on new cohorts. To support safe use, a data distribution check that compares user-provided input data with the training data distribution is provided in the reports. Where substantial differences are detected, the user is presented with a warning that model predictions may be less reliable and that local validation or further assessment may be required before interpretation.

### Potential workflow integration and stewardship implications

The intended role of the BC outcome prediction model is as an adjunctive decision-support tool within a broader diagnostic stewardship workflow, rather than as an autonomous rule for withholding BCs or antimicrobial therapy. In a possible implementation, routinely available CBC/DIFF or CBC/DIFF/CPD results would be processed once available from the haematology analyser, and the predicted probability of BC positivity could be returned to a laboratory information system, electronic medical record or stewardship dashboard. The prediction could then support review of BC ordering patterns, prioritization of patients for clinical or antimicrobial stewardship review or local audit of diagnostic practice.

Importantly, prediction alone does not directly reduce antimicrobial use or AMR. Any stewardship impact would be indirect and dependent on the way the model is embedded into clinical workflows, the thresholds used, the availability of clinician review and the governance processes surrounding its use. A high predicted probability may support escalation, prioritized review or increased diagnostic vigilance. Conversely, a low predicted probability should not be used in isolation to withhold BCs or antimicrobial therapy, particularly in patients with suspected sepsis, haemodynamic instability, immunocompromise or other high-risk clinical features.

The safety implications of false-negative predictions are, therefore, central to any clinical implementation. A false-negative prediction could delay BC collection, delay recognition of BSI or provide false reassurance if interpreted without clinical context. For this reason, local validation, prospective evaluation, conservative threshold selection, clinician override and ongoing monitoring of false-negative cases would be required before use in clinical decision-making. The potential relationship between this pipeline and antimicrobial stewardship should, therefore, be understood as enabling reproducible development and evaluation of prediction models, rather than demonstrating that the resulting models reduce antimicrobial exposure or AMR.

### Limitations

Several limitations should be noted. First, the pipeline currently supports only binary classification tasks; multi-class extensions would require modifications to the evaluation metrics and class weighting strategy. Second, hyperparameter optimization is not implemented in the current version, as the pipeline is designed to establish baseline models. Integration of hyperparameter search within the nested CV framework is a potential area for future development. The default model parameters presented were selected as conservative baseline settings. Third, the clinical utility of the exported models depends on the quality and representativeness of the training data. External validation on independent cohorts in different clinical settings and analyser configurations is essential before clinical deployment. The current pipeline also assumes that each row in the input dataset represents an independent prediction instance. It does not currently implement patient-level or admission-level grouping during CV. Therefore, when datasets contain multiple cultures from the same patient or repeated admissions, users should preprocess the data before using the software to ensure that the chosen unit of analysis is appropriate and that related samples are not split across training and evaluation partitions. Failure to account for repeated observations may result in optimistic performance estimates. Future versions of the software will aim to support group-aware splitting using patient-level or admission-level identifiers. The pipeline neither enforces nor proposes any data preparation approaches. The assumption is that the data has been appropriately prepared by the researcher before utilizing this model training evaluation pipeline. Finally, this manuscript presents a software pipeline for training, evaluation, reporting and deployment of BC outcome prediction models; it does not demonstrate that use of the resulting models reduces BC overuse, antimicrobial exposure or AMR. These outcomes would require prospective implementation studies that evaluate how predictions are incorporated into clinical workflows, how clinicians respond to model outputs and whether patient safety is maintained, particularly with respect to false-negative predictions.

## Conclusion

The software presented in this paper is an open-source, configurable ML pipeline that addresses a key limitation in the literature regarding transparent methodological approaches to BC outcome classification, presented to the wider community in a software-first approach. We present the integration of established modelling, evaluation, reporting and artefact-export methods into a reproducible software workflow tailored to microbiology and infection science applications. By implementing nested CV with feature selection inside the evaluation loop, the pipeline produces unbiased performance estimates. The export of LR coefficients and DT rules in portable formats enables deployment in clinical environments without a Python runtime. The configurable architecture allows adaptation to arbitrary binary classification problems in infectious disease without source code modification. This software is available under the MIT licence and is designed to support both research and clinical decision support applications in diagnostic stewardship, assuming local validation and compliance with regulatory standards have been met.

## Abbreviations

AMR: antimicrobial resistance
AUC: area under the curve
BC: blood culture
BSI: bloodstream infection
CBC: complete blood count
CPD: cell population data
CV: cross-validation
DIFF: white blood cell differential
DT: decision tree
LR: logistic regression
ML: machine learning
MLR: monocyte-to-lymphocyte ratio
NLR: neutrophil-to-lymphocyte ratio
RF: random forest
RFE: recursive feature elimination
SHAP: Shapley additive explanation
XG: XGBoost
